# Trends in Hip Fracture Incidence in Japan: Estimates Based on Nationwide Hip Fracture Surveys From 1992 to 2017

**DOI:** 10.1002/jbm4.10428

**Published:** 2020-11-30

**Authors:** Eri Takusari, Kiyomi Sakata, Tsutomu Hashimoto, Yasumasa Fukushima, Toshitaka Nakamura, Hajime Orimo

**Affiliations:** ^1^ Department of Hygiene and Preventive Medicine Iwate Medical University School of Medicine Iwate Japan; ^2^ Wakayama Medical University Wakayama Japan; ^3^ Ministry of Health, Labour and Welfare Tokyo Japan; ^4^ Touto Sangenjaya Rehabilitation Hospital Tokyo Japan; ^5^ Japan Osteoporosis Foundation Tokyo Japan

**Keywords:** AGING, HIP FRACTURE INCIDENCE, JAPAN, OSTEOPOROSIS

## Abstract

As of 2015, the aging population in Japan was the largest in the world. Although the National Database of Health Insurance Claims (NDB) was developed in 2012, long‐term trends regarding hip fracture incidence in Japan remain unclear. In order to clarify the trend in hip fracture incidence from 1992 to 2017, we estimated the number of new hip fractures in 2017, the seventh in a series of nationwide hip fracture surveys performed every 5 years since 1987. We also investigated regional differences in hip fracture incidence. We collected data through a nationwide mail‐in survey of orthopedic institutions in Japan and calculated hip fracture incidence by sex and age, as well as standardized incidence ratio (SIR) across 12 districts. The total number (95% confidence interval) of hip fractures in 2017 was estimated at 193,400 (187,300–199,500), occurring in 44,100 (42,700–45,500) males and 149,300 (144,500–154,100) females. Of all the hip fracture surveys from 1992 to 2017, the 5‐year hip fracture increase rates from 2012 to 2017 was the lowest among female patients. In males, the 5‐year rates from 2012 to 2017 were lower than those from 2007 to 2012. The age‐adjusted incidence rates for patients in both sexes did not show significant change in the 25‐year period. The estimated incidence rates in 2017 for patients aged 70 to 79 years in both sexes were lowest from 1992 to 2017, and declined significantly over the 25‐year period. SIRs differed between northeast and southwest regions. Our findings were similar to those from a previous study in Japan using the NDB from 2012 to 2015. Progress in the development of osteoporosis medication may contribute to the continuous decline in the 70‐year to 79‐year age group. © 2020 American Society for Bone and Mineral Research © 2020 The Authors. *JBMR Plus* published by Wiley Periodicals LLC on behalf of American Society for Bone and Mineral Research.

## Introduction

As of 2015, Japan had the largest aging population, not only in Asia but also in the world.^(^ ^1^ ^)^ Hip fractures contribute to complications of osteoporosis such as mortality, morbidity, and increased economic costs, given the aging global population.^(^ ^2^, ^3^ ^)^ Worldwide projections for hip fractures performed in the 1990s calculated a marked increase in the total number of hip fractures by 2050, half of which were expected to occur in Asia.^(^ ^4^, ^5^ ^)^ The Asian Federation of Osteoporosis Societies (AFOS) projects that the Asian countries will experience an 2.28‐fold increase in hip fracture frequency from 2018 to 2050.^(^ ^6^ ^)^

Hip fracture incidences declined in Europe, North America, and Oceania especially since the 1990s,^(^ ^7^ ^)^ and changed downward in Taiwan from 2003.^(^ ^8^ ^)^ There is global variation in hip fracture rates and in 10‐year probability of major osteoporotic fractures, rates in Japan are higher than those in other Asian countries, and are similar to those in Europe and North America.^(^ ^9^ ^)^

Although the National Database of Health Insurance Claims (NDB) was developed in 2012 and Tamaki and colleagues^(^ ^10^ ^)^ reported that although no change was noted in females, the age‐standardized hip fracture incidence in males increased significantly in Japan from 2012 to 2015, and long‐term trends regarding hip fracture incidence in Japan remain unclear.

In order to study the risk of fracture in the rapidly aging Japanese population, we have conducted nationwide hip fracture surveys every 5 years from 1987. The first survey in 1987 started with 53,200^(^ ^11^ ^)^ new hip fracture patients and set out to examine the incidence of hip fracture in this group of patients by sex and age. In the second survey, in 1992, we improved the accuracy of nationwide estimates and calculated the number of new patients to be 76,600.^(^ ^12^ ^)^ In the third survey, in 1997, the number was estimated to be 92,400^(^ ^13^ ^)^; in the fourth survey, in 2002, 117,900^(^ ^14^ ^)^; in the fifth survey in 2007, 148,100^(^ ^15^ ^)^; and in the sixth survey in 2012, 175,700.^(^ ^16^ ^)^

The present study is the seventh survey of patients with hip fracture. In this study, we aimed to calculate the number of new patients with hip fracture in 2017, to analyze the trends in incidence during the 25‐year period from 1992 to 2017, and to highlight any regional differences in hip fracture incidence in Japan.

## Materials and Methods

The sampling method, the questionnaire administered, and the estimation formulas were the same as the previous survey.^(^ ^16^ ^)^

### Sampling

The subjects of this study were hospitals and clinics with beds including or specializing in orthopedics throughout Japan. To calculate the number of new patients with hip fracture in 2017, we divided hospitals and clinics into 13 strata according to the number of beds (Table 1), ensuring comparability with past surveys.^(^ ^12^ ^)^ For a nationwide estimate, all hospitals with 200 beds or more were included and hospitals or clinics with less than 200 beds were selected randomly using Neyman's allocation method^(^ ^17^ ^)^ to minimize standard error the same as that used in past surveys since 1992.^(^ ^12^ ^)^ For regional estimates, we used all hospitals with 20 beds or more and used Neyman's allocation method to randomly select clinics with less than 20 beds in order to improve estimation accuracy. The abovementioned sampling method is the same as that used in past surveys since 2002.^(^ ^14^ ^)^ In this study, 4000 institutions were selected for a nationwide estimate and 5037 were selected for regional estimates among all 6495 orthopedic institutions in Japan based on hospital data from Wellness Co., Ltd. (Table 1).

**Table 1:** Number of Institutions Including Orthopedics in Japan and Sampling Numbers for a Nationwide Estimate and Regional Estimates According to the Number of Beds

Stratum	Beds (*n*)	Institutions (*n*)	Sampling for nationwide estimate *n* (%)	Sampling for regional estimate *n* (%)
1	≤19	1685	227 (13.5)	227 (13.5)
2	20–49	374	180 (48.1)	374 (100)
3	50–99	1202	723 (60.1)	1202 (100)
4	100–149	814	511 (62.8)	814 (100)
5	150–199	795	734 (92.3)	795 (100)
6	200–299	555	555 (100)	555 (100)
7	300–399	450	450 (100)	450 (100)
8	400–499	273	273 (100)	273 (100)
9	500–599	133	133 (100)	133 (100)
10	600–699	90	90 (100)	90 (100)
11	700–799	43	43 (100)	43 (100)
12	800–899	30	30 (100)	30 (100)
13	900+	51	51 (100)	51 (100)
	Totals	6495	4000 (61.6)	5037 (77.6)

### Questionnaire

We mailed a questionnaire to all selected institutions requesting the number, sex, and age of new patients treated for hip fracture between January 1 and December 31 of 2017. In order to maintain consistency, our definition of hip fracture as femoral neck, intertrochanteric, and subtrochanteric fracture was the same as the definition used in the previous surveys. To avoid double‐counting new hip fracture patients, we excluded patients that underwent surgery for hip fracture at other hospitals and patients undergoing rehabilitation.

The study protocol was approved by the Ethics Committee of the Iwate Medical University School of Medicine. We only asked for the frequency of hip fracture in each facility and the sex and age of each patient, all data were anonymous. Due to the retrospective nature of our study and the anonymity of the data, the need for informed consent was waived.

### Estimation formula

For nationwide estimates, we calculated the number of new patients with hip fracture as follows:

$$\;\text{Number of patients}=\sum \left(\frac{\mathrm{Ni}}{\mathrm{ni}}・\mathrm{Pi}\right)\;,$$

(1)where *Ni* is the number of institutions in each stratum, *n*_*i*_ is the number of institutions' responses, and *P*_*i*_ is the sum of the number of new patients in each stratum.^(^ ^12^ ^)^ We calculated each 5‐year hip fracture increase rates^(^ ^16^ ^)^ as follows: 5‐year hip fracture increase rates = 5‐year hip fracture increase number ÷ previous number of hip fractures × 100. We used 2015 national census data^(^ ^18^ ^)^ to calculate the nationwide hip fracture incidence, and the population acquired from 2010 world population prospects from the United Nations^(^ ^19^ ^)^ to calculate age‐adjusted incidences. Trends in estimated incidence were tested by executing single regression analyses, and a *p* < .05 was regarded as statistically significant.

For regional estimates, we divided the land into the following 12 districts according to the National Health and Nutritional Survey in Japan: Hokkaido, Tohoku, Kanto I, Kanto II, Hokuriku, Tokai, Kinki I, Kinki II, Chugoku, Shikoku, Northern Kyushu, and Southern Kyushu. We estimated each regional standardized incidence ratio (SIR) using the following equation: SIR$=\frac{B}{\sum \left(I\times P\right)\;}$, (2)where *B* is the estimated number of patients in each district, *I* is the nationwide hip fracture incidence by sex and age stratum, and *P* is the population in the district by sex and age stratum.^(^ ^12^ ^)^ Because the denominator was the expected number of patients, we calculated the SIR as the ratio of estimated number to expected number of patients with hip fracture, using 2015 national census data by district.^(^ ^18^ ^)^

All analyses were performed using SAS version 9.4 (SAS Institute, Inc., Cary, NC, USA).

## Results

### Respondents

We achieved a response rate of 61.4% for our nationwide estimate, with 2454 replies from 4000 sampled institutions. Institutions with 800 to 899 beds and those with less than 19 beds had a response rate higher than 70%, whereas the lowest response rate (51.1%) was from institutions with 500 to 599 beds. For regional estimates, 3027 institutions replied to our survey, for a response rate of 60.1% (Table 2). The regional response rates were 68.0%, 73.4%, 55.1%, 61.3%, 68.1%, 58.3%, 54.3%, 55.4%, 60.3%, 63.5%, 61.6%, and 59.8% in Hokkaido, Tohoku, Kanto I, Kanto II, Hokuriku, Tokai, Kinki I, Kinki II, Chugoku, Shikoku, Northern Kyushu, and Southern Kyushu, respectively.

**Table 2:** Number of Responding Institutions and Response Rates for Nationwide and Regional Estimates According to the Number of Beds

		For nationwide estimate		For regional estimate	
Stratum	Beds (*n*)	Sampling (*n*)	Responding and response rate *n* (%)	Sampling (*n*)	Responding and response rate *n* (%)
1	≤19	227	163 (71.8)	227	163 (71.8)
2	20–49	180	115 (63.9)	374	241 (64.4)
3	50–99	723	453 (62.7)	1202	708 (58.9)
4	100–149	511	320 (62.6)	814	486 (59.7)
5	150–199	734	455 (62.0)	795	481 (60.5)
6	200–299	555	316 (56.9)	555	316 (56.9)
7	300–399	450	261 (58.0)	450	261 (58.0)
8	400–499	273	161 (59.0)	273	161 (59.0)
9	500–599	133	68 (51.1)	133	68 (51.1)
10	600–699	90	61 (67.8)	90	61 (67.8)
11	700–799	43	24 (55.8)	43	24 (55.8)
12	800–899	30	22 (73.3)	30	22 (73.3)
13	900+	51	35 (68.6)	51	35 (68.6)
	Total	4000	2454 (61.4)	5037	3027 (60.1)

### Nationwide estimates

**Fig 1:**
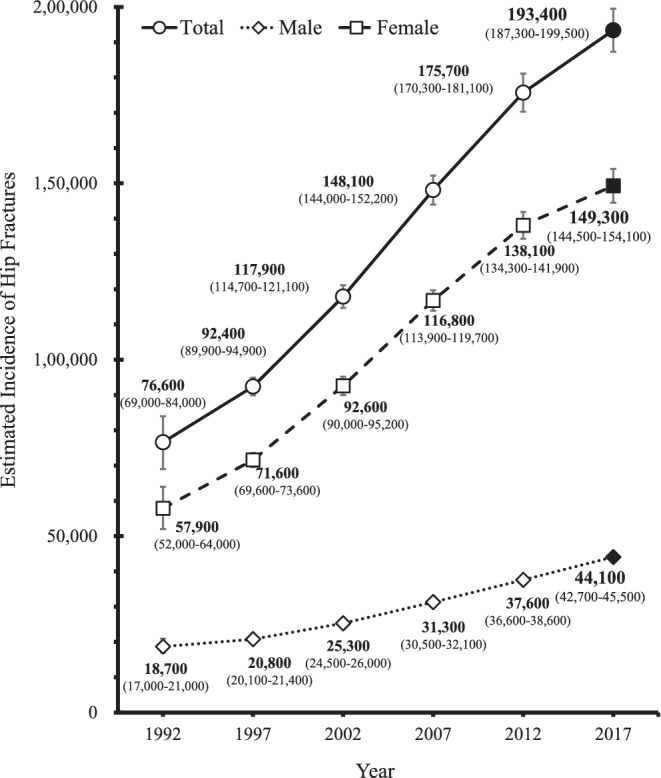
Estimated number (95% CI) of hip fractures by sex, 1992–2017.

The trends in the estimated number of hip fractures are presented in Fig. 1. The total number (95% confidence interval [CI]) of hip fractures in 2017 was estimated at 193,400 (95% CI, 187,300–199,500), occurring in 44,100 (95% CI, 42,700–45,500) males and 149,300 (95% CI, 144,500–154,100) females. Of all the hip fracture surveys from 1992 to 2017, the 5‐year hip fracture increase rates from 2012 to 2017 were the lowest in terms of total (+10.1%) and female (+8.1%) patients. When comparing the last two surveys, from 2007 to 2012 and 2012 to 2017, the 5‐year hip fracture increase rates were lower in all patients (+10.1%), males((+17.3%), and females(+8.1%) in the latter time period compared to the former time period at (+18.6%), (+20.1%), and (+18.2%), respectively.^(^ ^16^ ^)^

Table 3 shows the estimated annual hip fracture incidence per 100,000 by sex and age from 1992 to 2017. In the 25‐year period, the crude incidence rates for patients all ages in both sexes increased, whereas the age‐adjusted incidence rates in both sexes showed no significant change. The incidence rates for males aged 80 to 89, for females aged 40 to 49 and patients aged ≥90, in both sexes, significantly increased from 1992 to 2017, whereas the incidence rates for patients aged 70 to 79 years in both sexes, showed a significant decline from 1992 to 2017 and were the lowest of all age groups for the 25‐year period.

**Table 3:** Trends in Estimated Incidence of Hip Fracture Per 100,000, 1992–2017

Category	1992	1997	2002	2007	2012	2017	*p* value for trend^a^
Male, age (years)							
≤39	3.6	3.0	3.0	3.2	2.9	3.3	0.476
40–49	10.3	9.1	8.4	9.2	10.9	10.6	0.416
50–59	22.1	20.0	18.2	20.3	22.3	26.9	0.218
60–69	57.4	51.2	52.6	48.1	50.3	57.6	0.872
70–79	191.3	172.9	174.9	181.2	168.8	156.5	0.045
80–89	560.2	574.1	586.1	610.3	608.1	606.5	0.009
90+	1249.6	1288.9	1413.9	1466.2	1594.6	1729.0	<0.001
All ages (crude)	30.8	33.8	40.8	51.1	61.0	73.6	<0.001
Age‐adjusted^b^	25.6	24.0	24.3	25.1	25.2	26.1	0.340
Female, age (years)							
≤39	1.6	1.3	1.2	1.5	1.4	1.2	0.390
40–49	6.1	6.0	5.8	7.0	7.3	7.6	0.017
50–59	28.2	23.9	24.1	29.5	31.3	36.7	0.068
60–69	96.9	90.7	91.1	81.1	86.6	94.9	0.562
70–79	443.2	408.5	410.7	397.1	367.1	315.5	0.005
80–89	1396.0	1477.9	1561.0	1571.4	1510.3	1392.1	0.909
90+	2646.6	2810.4	3155.2	3135.8	3232.5	3181.5	0.022
All ages (crude)	92.0	111.9	144.3	181.4	213.1	235.4	<0.001
Age‐adjusted^b^	49.0	48.1	49.9	49.8	48.6	46.0	0.289

a The *p* value for trend were calculated by executing single regression analysis.

b Age‐adjusted incidence were computed using population acquired from 2010 World Population Prospects from United Nations by 5‐year age group.

### Regional estimates

**Fig 2:**
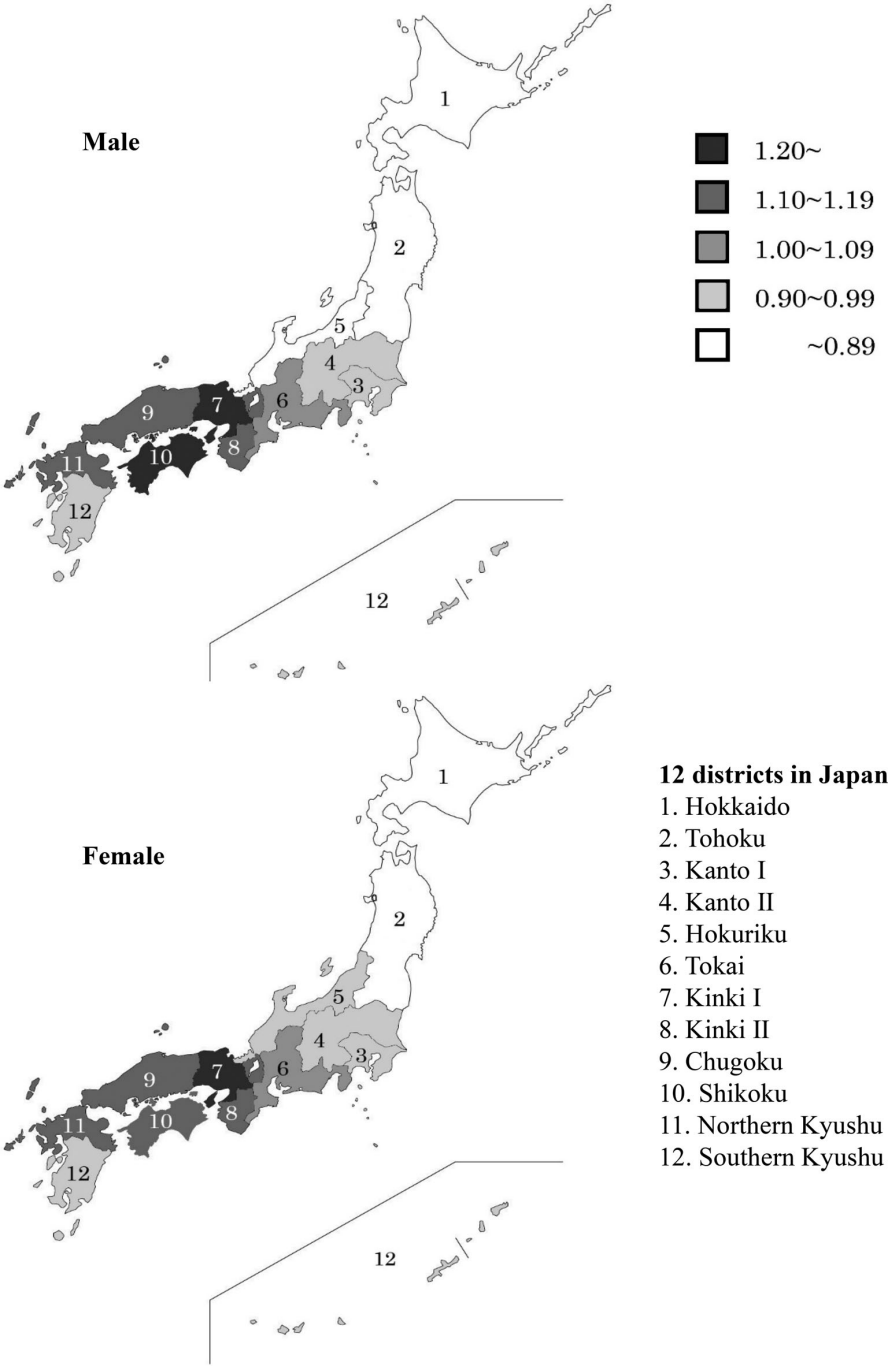
Standardized incidence ratio of hip fractures in 2017 by sex and district.

As shown in Fig. 2, regional differences were observed in hip fracture SIRs in 2017. Male SIRs were high in Kinki I (1.30) and Shikoku (1.21), and low in Tohoku (0.78), Hokkaido (0.82), and Hokuriku (0.87). Female SIRs were high in Kinki I (1.23) and low in Tohoku (0.74) and Hokkaido (0.86). The highest/lowest SIR ratios were 1.67 in males and 1.66 in females.

## Discussion

This study showed the trends in the number and incidence of hip fracture patients in Japan during population aging from 1992 to 2017. Despite an increase in the number of hip fracture patients, the 5‐year rate of increase from 2012 to 2017 was lower than that from 2007 to 2012, especially in females. The age‐adjusted incidence rates for patients in both sexes did not change in the 25‐year period; nevertheless, the incidence rates for patients aged 70 to 79 years in both sexes showed a significant decline from 1992 to 2017 and were the lowest of all age groups for the 25‐year period. Furthermore, our SIR results revealed northeast‐southwest regional differences in Japan.

The abovementioned findings, especially those for the period 2012 to 2017, are similar to the results of a previous Japanese study conducted using the NDB from 2012 to 2015.^(^ ^10^ ^)^ The variation in the estimated annual hip fracture incidence by sex and age from 2012 to 2017 resembles the trends reported from 2012 to 2015 using NDB, especially in males aged 70 to 79 years and females ≥70 years. Northeast‐southwest regional differences exist in both surveys despite differences in district classification. The main difference between the surveys is that our estimated number of hip fractures in 2012 was approximately 1.3 times higher than the number of events reported by the NDB in 2012.^(^ ^10^, ^16^ ^)^ Our estimated hip fracture frequency in 2012 was higher than that reported by the NDB in the same year because we included patients who were excluded from the NDB study, such as those with multiple fracture events, non‐operative patients, and patients aged <40 years. Hagino and colleagues^(^ ^20^ ^)^ reported that the annual incidence of second hip fracture was 3.4% in Japan. Surgical treatment was selected in 95.4% of patients and 4.6% underwent conservative treatment from 2009 to 2014 in hospitals or clinics approved by the Japanese Orthopedic Association (JOA) or the Japanese Clinical Orthopedic Association.^(^ ^21^ ^)^ Considering these excluded events, our estimated hip fracture incidence would inevitably exceed that derived from the NDB study in 2017.

On the other hand, the AFOS projected 179,202 hip fractures among people in Japan aged ≥50 years in 2018 and 242,990 in 2050,^(^ ^6^ ^)^ with the 2018 projection being lower than our estimated number of 193,400 in 2017. The AFOS used two data points: our previous survey^(^ ^16^ ^)^ and a study conducted in the Niigata prefecture.^(^ ^22^ ^)^ The standardized claims‐data ratios using the NDB in Niigata were relatively low in both sexes,^(^ ^10^ ^)^ our SIRs from the Hokuriku district—which includes the Niigata prefecture—were also relatively low in both sexes from 2002 to 2017.^(^ ^14^, ^15^, ^16^ ^)^ As the AFOS used data in the Niigata prefecture, the results might lack some generalizability. Therefore, the AFOS projection in Japan might be low due to this bias and because of the exclusion of patients aged <50 years.

The estimated incidence rates for aged 70 to 79 years in both sexes had the lowest rates in the 25‐year period from 1992 to 2017, and declined continuously in the 25‐year period. Additionally, the 5‐year rate of increase in the estimated number of hip fracture patients from 2012 to 2017 was lower than that from 2007 to 2012, mainly due to the number of females.

Both our sixth survey in 2012^(^ ^16^ ^)^ and the previous study using NDB from 2012 to 2015^(^ ^10^ ^)^ showed that hip fracture incidence rates decreased in elderly females, indicating that the use of bisphosphonates (BPs) might play a role in this trend. Several foreign studies^(^ ^8^, ^23^, ^24^, ^25^ ^)^ and one local Japanese study in Niigata^(^ ^22^ ^)^ have shown that the use of bisphosphonates contribute to a decrease in hip fracture incidence, especially in elderly females. The sale of BPs in Japan began in 1996, and sales grew from 2002 to 2011.^(^ ^26^ ^)^ The number of dosage forms for BPs increased and a generic formulation was released in 2011, and BPs sales stayed flat from 2012 to 2017.^(^ ^26^ ^)^ According to the NDB open database, the number of patients treated with BPs remained nearly constant in the 2015–2016 fiscal year.^(^ ^27^ ^)^ Furthermore, teriparatide (TPTD) and denosumab (Dmab) were placed on the Japanese market in 2010 and 2013, respectively. There is high‐level evidence that all three of these “anchor drugs” are effective for fracture prevention.^(^ ^28^, ^29^ ^)^ TPTD sales grew remarkably from 2010 to 2012,^(^ ^26^ ^)^ and the number of patients treated with TPTD slightly grew in the 2015–2016 year according to NDB open database.^(^ ^27^ ^)^ Additionally, despite low numbers of patients initially treated with Dmab in fiscal year 2015, the number dramatically increased the next fiscal year.^(^ ^27^ ^)^

Our estimates showed a significant decline in hip fracture incidence among patients in their 70s in both sexes. One explanation is the progress in the development of medication for osteoporosis in Japan, especially since 2010. For the 2017 fiscal year, most of the osteoporosis drug prescription were for patients in their 70s.^(^ ^27^ ^)^ Given the rapid increase in social security expenditures due to an aging population,^(^ ^30^ ^)^ fracture prevention is becoming an important issue in Japan. Kamata and colleagues^(^ ^28^ ^)^ were concerned about osteoporosis treatment in Japan, delayed treatment initiation, and small number of treated patients. Several Japanese studies have investigated cost‐effectiveness of osteoporosis screening, medication for osteoporosis, and osteoporosis liaison service in postmenopausal women.^(^ ^31^, ^32^ ^)^ However, because these studies were sex‐specific, the results are only relevant for females. Therefore, studies of osteoporosis screening and treatment in both males and females are warranted in order to prevent osteoporotic fractures in all patients.

This study has several limitations. First, we used a mail‐in survey. Although this was done in order to ensure methodological consistency among our previous surveys, this approach resulted in a response rate of only 61.4%, which was less than that obtained in the study using NDB. It was possible that the institutions that answered the survey treated more inpatients than the institutions who did not answer, because the orthopedists in middle‐sized institutions located in urban areas may have been too busy to reply because of many outpatients. Hence, our estimations may have been overestimated. Second, our survey only asked for the annual number of new patients with hip fracture and the sex and age of every patient; it did not confirm items asked by the JOA such as past medical history including osteoporosis, cause of fractures, or fracture type and treatment.^(^ ^21^ ^)^ Therefore, we could not investigate the direct association between osteoporosis medication and hip fracture incidence using our data. Third, if the patients' place of residence was far from their treatment facility, they may have been inaccurately captured by our district classifications. However, the possibility of district misclassification in our study is lower than that in the NDB study, because we created our 12 districts according to the National Health and Nutrition Survey, resulting in regions larger than the typical Japanese prefecture and thus likely to include people living far from their treatment center. Further studies should be conducted to clarify the incidence of hip fractures considering the patient's residence or home environment rather than their medical institution only.

In conclusion, the present study found that 2017 had the highest total number of fractures of all surveys from 1992 to 2017 although the incidence of hip fracture in those aged 70 to 79 years was lower than that found in our previous surveys. Furthermore, northeast‐southwest regional differences persisted across Japan.

## Author Contributions

**Eri Takusari:** Data curation; formal analysis; investigation; project administration; validation; visualization; writing‐original draft; writing‐review and editing. **Kiyomi Sakata:** Conceptualization; data curation; formal analysis; investigation; project administration; software; validation; visualization; writing‐review and editing. **Tsutomu Hashimoto:** Conceptualization; methodology; supervision; validation; writing‐review and editing. **Yasumasa Fukushima:** Methodology; supervision; validation; writing‐review and editing. **Toshitaka Nakamura:** Funding acquisition; resources; supervision; validation; writing‐review and editing. **Hajime Orimo:** Conceptualization; funding acquisition; methodology; resources; supervision; validation; writing‐review and editing.

### Peer Review

The peer review history for this article is available at https://publons.com/publon/10.1002/jbm4.10428.
